# Supradiaphragmatic intrathoracic migration of ventriculoperitoneal shunt with “double bending sign”

**DOI:** 10.1016/j.radcr.2022.05.002

**Published:** 2022-05-29

**Authors:** Shunta Tsuchida, Joji Tokugawa, Takamitsu Banno, Takashi Mitsuhashi, Makoto Hishii

**Affiliations:** a Clinical Training Center, Juntendo University Nerima Hospital, Tokyo, Japan; b Department of Neurosurgery, Juntendo University Nerima Hospital, Tokyo, Japan; c Department of General Thoracic Surgery, Juntendo University Nerima Hospital, Tokyo, Japan

**Keywords:** Cerebrospinal shunt, Complication, Hydrocephalus, Reoperation, Surgical injury

## Abstract

Ventriculoperitoneal shunt (VPS) is a common treatment for hydrocephalus. An 80-year-old woman presented with subarachnoid hemorrhage caused by rupture of an aneurysm of the right middle cerebral artery. Emergency clipping was performed. Hydrocephalus occurred shortly after and VPS placement was performed. She improved and was transferred to a rehabilitation hospital. She presented with dyspnea 5 months later. Chest computed tomography (CT) showed extensive pleural effusion and intrathoracic migration of the distal VPS catheter. Chest CT confirmed that the distal catheter had penetrated into the pleural cavity under the second rib, and the catheter tip was located at the bottom of the right thoracic cavity. Review of chest CT right after the shunt surgery found the distal catheter passing only under the second and third ribs and otherwise located in the subcutaneous layer to the abdominal cavity. Chest radiography showed that the distal shunt tube was distorted in a characteristic “double bending sign.” This rare case of supradiaphragmatic intrathoracic migration of VPS indicates a possible mechanism of this migration, based on the anatomical physiology, and that “double bending sign” indicates the need for further investigation.

## Introduction

Intrathoracic migration of the distal ventriculoperitoneal shunt (VPS) catheter is a rare complication. We report a case of supradiaphragmatic intrathoracic migration of the distal VPS catheter and discuss the mechanism, radiographical characteristics, and literature review.

## Case report

**Fig. 1:**
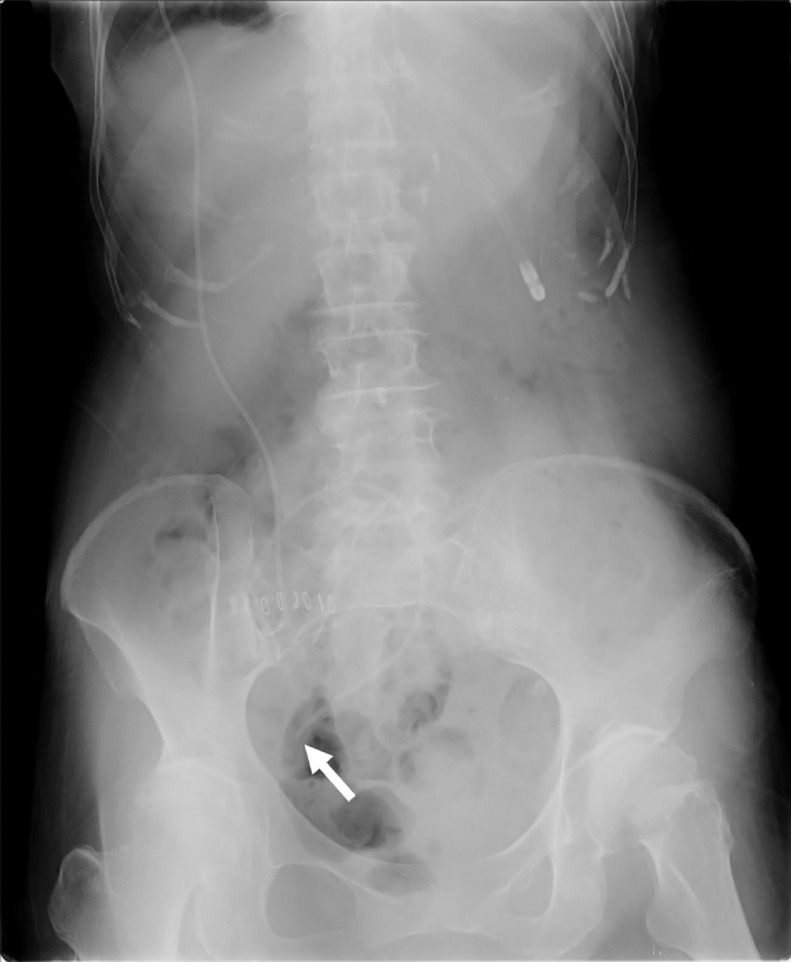
Abdominal radiograph taken right after the first ventriculoperitoneal shunt showing that the distal catheter is in the correct position. The white arrow indicates the tip of the catheter.

An 80-year-old woman presented to the emergency room with sudden onset of severe headache. Neuroimaging revealed subarachnoid hemorrhage caused by rupture of an aneurysm of the right middle cerebral artery. Emergency clipping surgery was performed. She developed hydrocephalus and right VPS was performed using a Codman CERTAS Plus Programmable Valve (Integra LifeSciences, Mansfield, MA) 4 weeks later (Fig. 1), then she was transferred to a rehabilitation hospital. Chest radiography was performed several times in the rehabilitation hospital. Chest radiography on day 115 after the first VPS revealed that the distal VPS catheter was looping in the right lung field, but this abnormality was not reported at the time. Five months after the transfer, she presented with dyspnea and chest computed tomography (CT) showed extensive pleural effusion and intrathoracic migration of the distal VPS catheter. The patient was transferred to our hospital for further investigation and treatment.

**Fig. 2:**
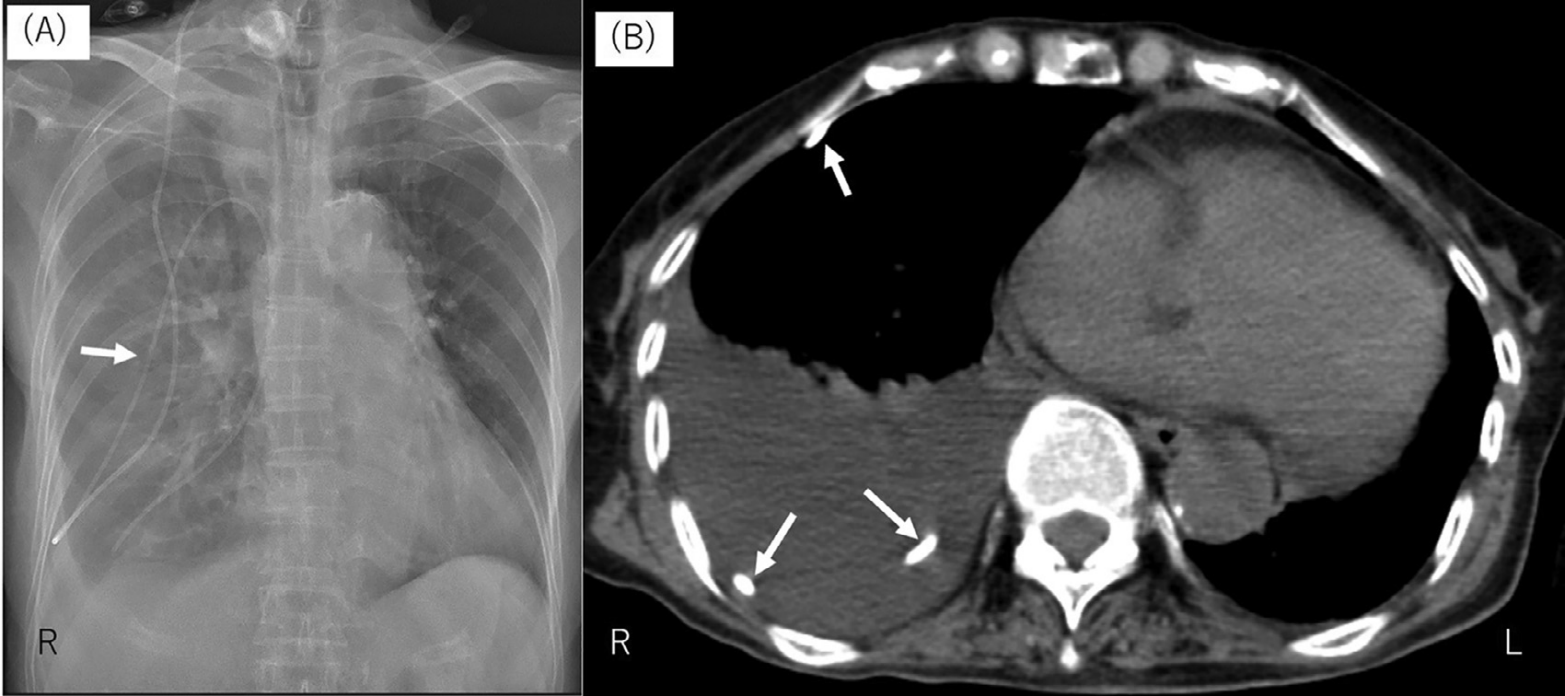
Chest radiograph (A) and computed tomography scan (B) taken on second admission to our hospital, on day 156 after the first ventriculoperitoneal shunt procedure, showing massive pleural effusion and abnormal position of the distal catheter (white arrows) in the right lung field.

**Fig. 3:**
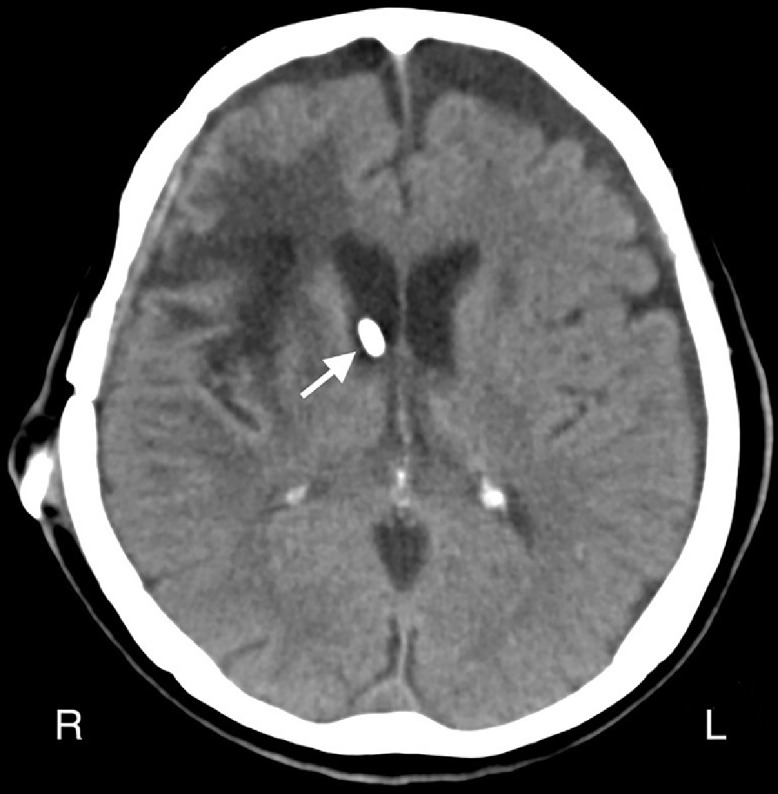
Head computed tomography scan taken on second admission to our hospital, on day 156 after the first ventriculoperitoneal shunt procedure, showing the tip of the ventricular tube (white arrow) in the right lateral ventricle.

**Fig. 4:**
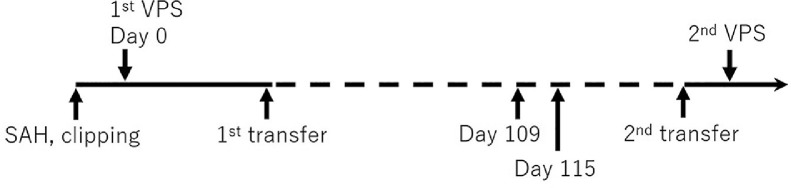
Chronological time course from the onset of subarachnoid hemorrhage (SAH) to the second ventriculoperitoneal shunt (VPS) procedure. The periods of hospitalization in our hospital and the rehabilitation hospital are expressed as the solid line and the dashed line, respectively.

Chest examination revealed decreased breathing sound in the right chest. Laboratory findings showed white blood cell count was 4.8 × 10^9^/L and C-reactive protein level was 0.18 mg/dL, but no inflammatory indicators suggesting pneumonia or shunt infection. Chest radiography and CT showed a large amount of pleural effusion and migration of the distal VPS catheter coiling in the right thoracic cavity (Fig. 2). Head CT showed no hydrocephalus and the ventricular catheter in the normal position (Fig. 3). Right thoracentesis and chest tube insertion were performed. Chest CT confirmed that the distal VPS catheter had penetrated into the pleural cavity under the second rib, and the catheter tip was located at the bottom of the right thoracic cavity. No catheter remained in the abdominal cavity. Revision of the right VPS was carried out: the catheter distal to the valve shunt was removed, and a new catheter was placed in the abdominal cavity. After the surgery, the patient's general status improved gradually, and she was again transferred to the rehabilitation hospital. The time course of the events is shown in Figure 4.

## Discussion

### Possible migration mechanism

**Fig. 5:**
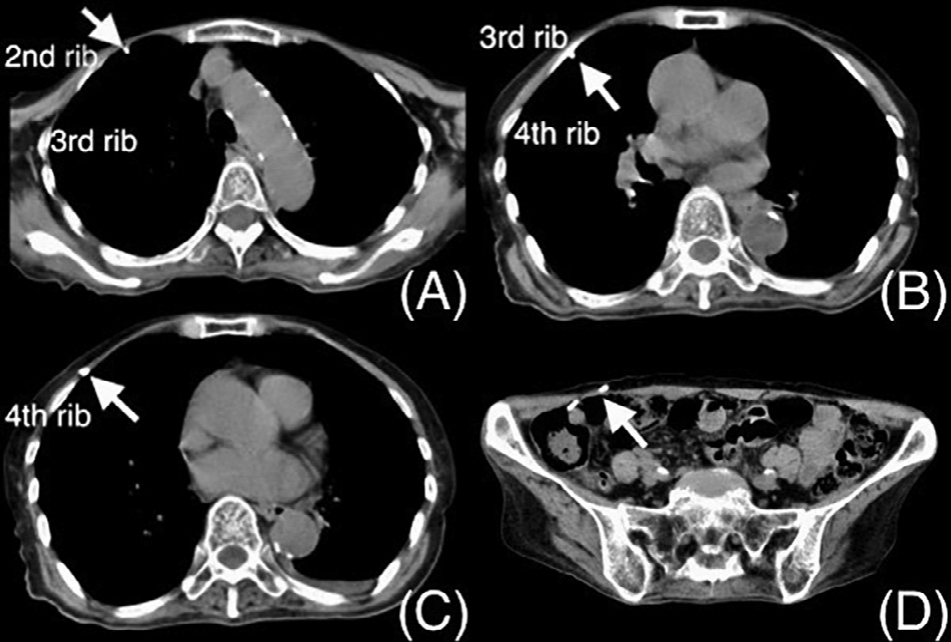
Chest and abdominal computed tomography scans taken 13 days after the first ventriculoperitoneal shunt procedure showing the distal catheter (white arrows) passing only under the second rib (A) and third rib (B), and over the fourth rib in the subcutaneous layer (C), and in the abdominal space (D).

**Fig. 6:**
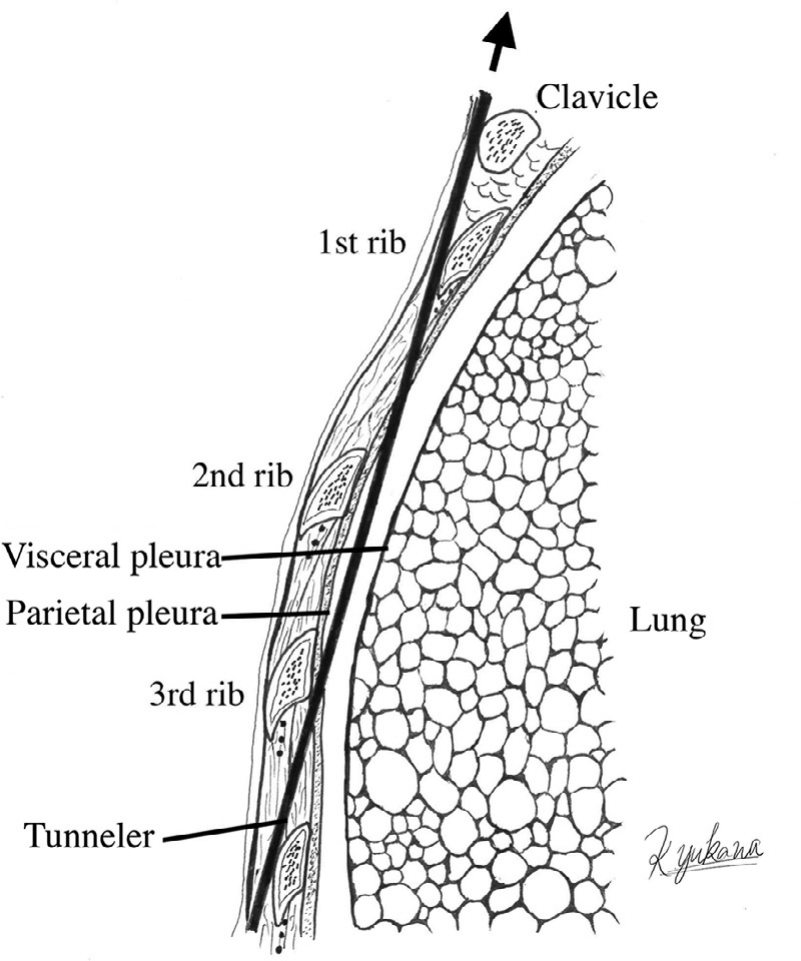
Possible course of the tunneler (thick black line) seen from the side. The black arrow indicates the direction of tunneling. The tunneler penetrates the intercostal muscle and parietal pleura at the third intercostal space, passes under the second and the third ribs, and proceeds over the first rib and the clavicle.

In our institute, the metal tunneler for the distal VPS catheter is always inserted through the subcutaneous layer of the abdominal wound in the cranial direction. In the present case, chest CT 13 days after the first VPS revealed that the distal catheter had passed only under the second and third ribs and was otherwise located in the subcutaneous layer of the abdominal cavity, which we did not detect at the time (Fig. 5). This postoperative image suggests that the tunneler was guided beneath the third rib and returned to the subcutaneous layer after passing beneath the second rib with minimum injury to the parietal pleura and without damaging the intercostal arteries, veins, or nerves (Fig. 6). This misguidance can be explained in 2 ways based on specific features of the anatomical structures. First, viewed from the side, the thorax has a gentle upward slope from the abdomen to the third rib, and a downward slope from the third rib toward the first rib and clavicle. Therefore, a straight metallic device (tunneler) will tend to follow a shorter route at the head of the slope; in other words, will easily pass under the ribs. Second, the “pump-handle movement” of the upper ribs during inspiration causes the lower part of the ribs to float upward. Therefore, the tunneler inserted from the caudal side can easily pass beneath the upper ribs.

The patient showed no respiratory distress intra- and postoperatively despite the position of part of the distal VPS catheter in the thoracic cavity under the second and third ribs, and the postoperative chest radiography showed no pneumothorax. The chest radiography confirmed that the distal VPS catheter was in the normal position until day 109 after the first VPS procedure, but the free end of the catheter was found in the thoracic cavity on day 115. Normal intrathoracic pressure is -8 cm H_2_O to -2 cm H_2_O at rest, and the internal pressure difference was about 40 cm H_2_O at maximum inspiration and expiration [1]. The patient had never smoked with no thorax deformity and was considered to have normal respiratory function. Our hypothesis for the migration is that constant and continuous negative intrathoracic pressure pulled on the intrathoracic part of the distal catheter and eventually drew the whole free end of the distal catheter into the thoracic cavity.

## Literature review

Complications of VPS can be classified as mechanical or nonmechanical. Mechanical complications include catheter obstruction, disconnection, and migration. Nonmechanical complications include infection and distal compartment-related complications such as pseudocyst formation, ascites, or pleural effusion. Catheter migration is a type of mechanical migration and can occur at various organs or sites as follows: ventricle, subgaleal space, breast, chest wall, heart, abdominal wall, genital organs, urinary organs, colon, liver, and combinations of these sites [2].

Thoracic migration of VPS can be classified into 3 types: (1) Intraoperative traumatic placement of a shunt, (2) migration into the chest by supradiaphragmatic or transdiaphragmatic route, and (3) plural effusion accompanying cerebrospinal fluid ascites [3]. The present case is classified as type 2), and only 6 similar cases of supradiaphragmatic intrathoracic migration of the distal catheter have been reported (Table 1) [2], [3], [4], [5], [6], [7]. This complication can happen at any age, as 4 patients (Cases 1-4) were neonates or infants, and 2 (Cases 5 and 6) were adults. The direction of the tunneling was “downward” (head to abdomen) in Case 3, which is opposite to “upward” in our case, but otherwise was not clearly described. Case 6 was likely “downward” because the catheter penetrated from the supraclavicular fossa. These findings suggest that this complication can occur with either direction of tunneling.

**Table 1:** Reported cases of supradiaphragmatic intrathoracic migration.

Cases	Authors	Age, sex primary disease	Time interval	Symptoms	Chest X-ray findings	CT findings	Entry site to the thoracic cavity
1	**Obrador and Villarejo, 1977**	4-month boy Aqueductal stenosis	13 months	Respiratory problem	Hydrothorax on the right side	Not mentioned	Not mentioned
2	**Dickman, et al. 1989**	1-month boy Myelomeningocele	5 days	Acute pallor, Tachypnea, Bradycardia, Irritability, Newly sunken fontanel	Pleural effusion, Mediastinal shift	Not mentioned	Not mentioned
3	**Johnson and Maxwell, 1995**	6.5-month boy Bulging anterior fontanel	4.5 months	Nasal flaring, Head bobbing, Intercostal retraction	Pleural effusion, Mediastinal shift	Not mentioned	Under the second rib and then over the third rib
4	**Saha, et al. 2018**	1-month boy Hydrocephalus with dorsal meningocele	5 years	Asymptom	No pleural effusion	Confirmed intrathoracic	Supra clavicular fossa
5	**Doh, et al. 1995**	52-year-old man Caroticocavernous fistula	3 years	Chest pain, Dyspnea	Pleural effusion	Incorrect location of the shunt under the ribs	Under the rib (not mentioned further)
6	**Rahami Rad, et al. 2007**	51-year-old woman Arachnoid cyst	4 months	Chest pain and dyspnea	Pleural effusion, Coiling in the chest	Passed in the supraclavicular fossa	Supraclavicular fossa

Pneumothorax right after VPS can develop with the same type of complication [8]. Pneumothorax probably results with just a slight difference in the angle that the tunneler penetrates the parietal pleura. Palpating the tip of the tunneler at all times is mandatory, although breast or obesity tissue sometimes deceives the surgeon [8].

## “Double bending sign”

**Fig. 7:**
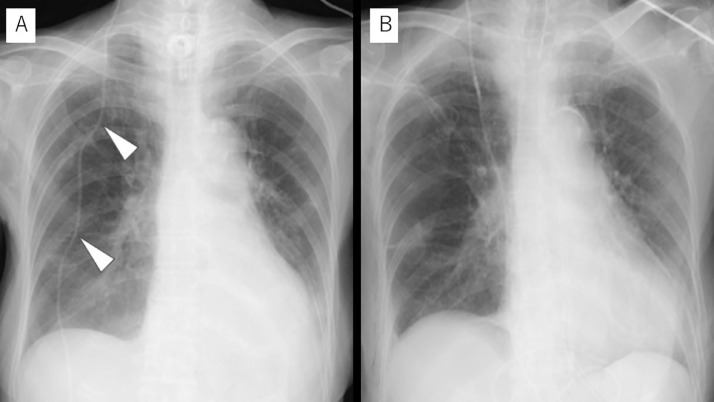
Chest radiographs after the first ventriculoperitoneal shunt procedure (A) and after the shunt revision (B). Note that the tube bends twice at the chest wall (white arrowheads), which we call the “double bending sign” on (A), whereas the tube has no bend in (B).

Chest and abdominal radiography in the anteroposterior view, not CT, are taken after VPS to confirm that the catheter is placed in the correct position. However, radiography cannot clearly exclude misguided catheter as in the present case. Chest radiography in the lateral view may help to detect such abnormality, but the angle of the film should always be tangential to the catheter, which is exceedingly difficult. We realized that the distal catheter was bending twice at the chest wall based on the chest radiography in the anteroposterior view taken immediately after the first VPS. The bending sites are equivalent to the second and third ribs, where the catheter was misguided. We named this finding as the “double bending sign” indicating the need for further radiological confirmation of the catheter placement (Fig. 7). To confirm the usefulness of this sign, we reviewed the postoperative chest radiographs of the 52 most recent consecutive VPS patients. The 3 reviewers, a resident (first author, ST), a board-certified neurosurgeon (coauthor, JT), and a thoracic surgeon (coauthor, TB) unfamiliar with postoperative chest radiography of VPS, independently checked the 52 chest radiographs. All 3 observers agreed that no “double bending sign” was seen in any of the 52 chest radiographs. The distal catheter should be straight or gently curved and should not have unnatural bending at the chest wall. If the postoperative chest radiography shows the “double bending sign,” thoracic CT should be performed.

## Conclusion

This rare case of supradiaphragmatic intrathoracic migration of distal VPS catheter suggests that the possible mechanism involves anatomical characteristics. This rare complication may remain asymptomatic for a while, but the “double bending sign” on postoperative radiography indicates the need for further investigation.

## Patient consent

Written informed consent was obtained from the patient for publication of this case report and accompanying images.
